# Supplementary material for: Adverse maternofoetal outcomes associated with ionised calcaemia, total calcaemia, albuminaemia, and calcium supplementation in pregnancy: Analysis from a resource-limited setting
Source: PLoS One. 2022 Aug 1;17(8):e0271525. doi: 10.1371/journal.pone.0271525 (PMC9342720; doi:10.1371/journal.pone.0271525)
Supplement: Attachment — Submitted filename: RESPONSE TO REVIEWERS.docx [file pone.0271525.s002.docx]

**RESPONSE TO REVIEWERS COMMENTS ON PONE-D-21-33966**

Dear Editor/Reviewer,

We wish to thank you for the contributions you have been put in to strengthen this manuscript. We have taken time and gone through each point and have responded while making changes to the manuscript. The response to reviewers comments have been presented here in a tabular form (below). Also, the recommendations of the editor in terms of PLoS one guidelines, ethical statement and data availability statement have been addressed.

| **REVIEWER’S COMMENTS** | **RESPONSE** |
| --- | --- |
| This is an article discussing an association between pregnant maternal serum calcium, ionized calcium and fetal outcomes including anthropometric indecies and physiological status using Apgar scores (AS) at on and five minutes after birth. The authors successfully showed an association of ionized calcium and low birth weight, low AS scores, and brachial circumferences among 1,074 pregnant women. | Thank you for your observation. |
| However, the reason why the authors pay attention to serum calcium and fetal growth and no information how amount of calcium supplement or calcium daily intake had during each trimester as the authors discussed in the discussion part. | Thanks for this observation. We designed and developed a research protocol which we submitted as was published in PLoS One (1). The present manuscript provides a report of some of the research findings. While we do acknowledge the limits associated with the cross sectional design (stated in the limits), we seek to faithfully report the outcomes of the study as designed in the protocol and recommended by PLoS One editorial policies on open science.  This work has been divided into three major write ups which have all been submitted to PLoS One.  We have a manuscript evaluating calcium intake (in terms of calcium supplementation practices in pregnancy) and determines factors affecting this practice in pregnancy (given that it is recommended by the WHO to be systematic in LMIC). This was submitted on the 1st 0ct 2021 (PONE-D-21-31679).  The second manuscript from this registered protocol focused on evaluating the prevalence of ionised and total hypocalcaemia and associated socio-demographic, obstetric and nutritional factors using standard statistics. It also finds out how calcium supplementation variables like daily dose, duration, taking calcium with other supplements affect calcaemic states. This has already gone through revision (PONE-D-21-31669R1)  The present manuscript therefore targets the hypocalcaemia-associated outcomes. It evaluates the prevalence of some adverse maternofoetal outcomes and finds out associations between calcaemic states (ionised and total calcium), albumin and maternofoetal outcomes. We feel that mixing calcium intake/calcium supplementation into this manuscript does not help us respond to any of its objectives. This aspect has been dealt with in our first manuscript. |
| Moreover, they did not show mother’s nutritional status including BMI at baseline and body weight gain during pregnancy. Unless such information is not shown, clinicians could not identify who is at risk of low ionized calcium concentration from mother’s nutrition. | We thank the Reviewer for this point. As stated in the in the registered protocol, we were interested in evaluating nutritional **habits or behaviours** and how they influence calcaemic states (already addressed in the second manuscript). We acknowledge that the nutritional **status** of these women was not evaluated in our protocol**.**  As stated in the registered protocol, our study was cross sectional in design and did not aim at finding causative factors of low hypocalcaemia in pregnancy (which would ideally be done using a longitudinal design). Evaluation of risk factors associated with hypocalcaemia is addressed by the second manuscript.  We used a crude BMI which was gotten immediately after delivery. Measurement of overweight and obesity in pregnancy using BMI was not one of our objectives. We believed that the error associated with its evaluation immediately after birth was common in all included women and the trend could therefore still be useful.  We understand that criticisms of this sort greatly improves the study and lends another dimension of its utility. We have taken note of this and will incorporate the pertinent points in subsequent research on this important subject |
| Additionally, English writing seems poor to be edited by native English writers. | Thanks for your observation. The whole manuscript has been edited by a native English speaker. |
| The timing to take blood also must be shown whether before breakfast, fating period, or early morning to standardize serum calcium concentrations. | Thanks for your pertinent observation. Blood sample collection time for calcium assays was random and depended on the time at which the participant was included into the study as has been the case in similar studies (2–6). No particular fasting indications were given to participants before sample collection. However, as stated in the methods section, precautions were taken to make sure measured calcium levels best reflect the calcaemic states of the participants. |
| In conclusion, I feel that this article must show mother’s nutritional status and daily nutrients intake in whom serum ionized calcium is lower than cutoff value and English edits. | We want to thank the Reviewer for the time taken to assess the value of this study, which he greatly appreciates and therefore provided constructive feedback. We have taken note of some of the pertinent comments which, unfortunately, can only be adequately addressed in a new study (longitudinal). We plan to take these into consideration as part of our future study. |
| However, the authors’ eye point of ionized calcium with potential function of fetal cell development seems opening novel scientific door to faeto-maternal affect. | Thank you very much. |
| As my conclusion, I must ask major revision for the points above mentioned. Otherwise, this must be rejected. | Thanks for your recommendation. We have provided a point by point response to the comments and substantially revised the manuscript. We remain open should there be other issues to address prior to publication of the manuscript following the publication of its protocol. |
| **MINOR REVIEWER’S COMMENTS** | **RESPONSES** |
| 1. No explanations of abbreviations in all tables. | Thanks for your observations, the tables have been edited and all abbreviations removed. See table 1, 2 and 3. |
| 2. Why they measured brachial circumference? I feel this must be overestimated because of effect of long-term floating in yolk sac fluid before birth. | Thanks for the pertinent point. We agree with the Reviewer that this measurement might be overestimated. But our aim was not to define with exactitude the prevalence of low brachial circumference at birth. In addition, we needed the brachial circumference at birth which is standardly measured like that in the hospitals (7,8). Moreover, even with the error, the trend of variation with calcaemic states will still be detected (7,8). |
| 3. What contents of interview questionnaire and was it validated, although the authors described that questionnaires were validated but no references to explain that? | Thanks for your observations. The questionnaire used in this study was published with the registered protocol which has been cited in the manuscript. The sentence fraction “validated questionnaires” is only used in the first sentence of the data analysis section and describes retained questionnaires. To eliminate the ambiguity, the word has been replaced with “retained”. See page 9, paragraph 1. |
| 4. Line 212, “was” was duplicated in a single sentence. | Thanks for the observation. The error has been corrected. See line 217, paragraph 2, page 11. |
| 5. The part of the end of discussion must be divided to “Limitations of the study” like the other articles. | Thanks for your recommendation. The section “limits of the study” has been added. See paragraph 1, page 21 |
| 6. Line 387: the word of “cause-effect relationship” might be changed to causal association. | Thanks for your suggestion. It has been changed to causal. See paragraph 1, page 21 |

**References**

1.	Ajong AB, Kenfack B, Ali IM, Yakum MN, Aljerf L, Telefo PB. Hypocalcaemia and calcium intake in pregnancy: A research protocol for critical analysis of risk factors, maternofoetal outcomes and evaluation of diagnostic methods in a third-category health facility, Cameroon. PLoS One. 2020;15(11 November).

2.	Ajong AB, Kenfack B, Ali IM, Yakum MN, Telefo PB. Prevalence and correlates of low serum calcium in late pregnancy: A cross sectional study in the Nkongsamba Regional Hospital; Littoral Region of Cameroon. PLoS One. 2019;14(11).

3.	Bako B, El-Nafaty AU, Mshelia DS, Gali RM, Isa B, Dungus MM. Prevalence and risk factors of hypocalcemia among pregnant and non-pregnant women in Maiduguri, Nigeria: A cross-sectional study. Niger J Clin Pract. 2021;

4.	Gebreyohannes RD, Abdella A, Ayele W, Eke AC. Association of dietary calcium intake, total and ionized serum calcium levels with preeclampsia in Ethiopia. BMC Pregnancy Childbirth. 2021;21(1).

5.	Kumar A, Agarwal K, Devi SG, Gupta RK, Batra S. Hypocalcemia in Pregnant Women. Biol Trace Elem Res. 2010 Jul;136(1):26–32.

6.	Benali AI, Demmouche A. Calcium Deficiency among Pregnant Women and their Newborns in Sidi Bel Abbes Region, Algeria. J Nutr Food Sci. 2014;04(06):4–7.

7.	Shankar R, Ramarajan A, Rani S, Seshiah V. Anthropometric and Skin Fold Thickness Measurements of Newborns of Gestational Glucose Intolerant Mothers: Does it Indicate Disproportionate Fetal Growth? J Obstet Gynecol India. 2020;

8.	Tiruneh C, Teshome D. Prediction of Birth Weight by Using Neonatal Anthropometric Parameters at Birth in Finote Selam Hospital, Ethiopia. Pediatr Heal Med Ther. 2021;
