# Supplementary material for: Adverse maternofoetal outcomes associated with ionised calcaemia, total calcaemia, albuminaemia, and calcium supplementation in pregnancy: Analysis from a resource-limited setting
Source: PLoS One. 2022 Aug 1;17(8):e0271525. doi: 10.1371/journal.pone.0271525 (PMC9342720; doi:10.1371/journal.pone.0271525)
Supplement: Attachment — Submitted filename: Response to Reviewers.docx [file pone.0271525.s003.docx]

**RESPONSE TO REVIEWER’S COMMENTS**

We wish to appreciate once more the efforts of the editor and the reviewers to improve the quality of this manuscript. We have presented the response to each reviewer’s comment in the table below.

| **Comments** | **Response** |
| --- | --- |
| 1. Does the manuscript adhere to the experimental procedures and analyses described in the Registered Report Protocol?  If the manuscript reports any deviations from the planned experimental procedures and analyses, those must be reasonable and adequately justified.  Reviewer #1: No  Reviewer #2: Yes | Thank you for your response. Our manuscript partly adheres to experimental procedures and analyses reported in the registered protocol. All the data collection steps were respected from the number of participants, recruitment of participants, methods of biochemical assays, measurement of maternal foetal parameters and data analysis. No additional data were collected to be able to tackle the objectives set in this manuscript.  We reported a slight deviation in the methods where we explained why instead of considering only the Nkongsamba Regional Hospital, we had to include 4 major health facilities in the Nkongsamba Health District. This is explained in the manuscript with the reason. Also, instead of excluding a set of participants with hypocalcaemia-causing pathologies, we including all women who were apparently healthy in the study and this statement was included and justified. See methods,line 121-132 and line 138-141. Page 8. |
| 2. If the manuscript reports exploratory analyses or experimental procedures not outlined in the original Registered Report Protocol, are these reasonable, justified and methodologically sound?   A Registered Report may include valid exploratory analyses not previously outlined in the Registered Report Protocol, as long as they are described as such.  Reviewer #1: Partly  Reviewer #2: Yes | Thank you for your response. This manuscript was written out from one of the objectives of the registered protocol.  Objective 3: Determine the prevalence of hypertensive disorders in pregnancy among hypocalcaemic pregnant women in the NRH and describe hypocalcaemia-associated maternofoetal outcomes (with foetal outcomes evaluated assecondary outcomes) (1). We reformulated this objective to that described in the manuscript because of the links between albumin, total and ionised calcaemia. We were interested in finding out if associations existed between these factors and maternofoetal outcomes and if yes, which factors had the strongest association. This has been explained with the reason/justification in the last paragraph of the introduction. See page 6, line 111-115.  However, this did not affect any initial methods related to data collection reported in the original protocol. We did not collect any additional data to respond to the objectives of this manuscript.  As stated in the original protocol, we have also included in this revised version the prevalence of hypertension in pregnancy among hypocalcaemic women. See data analysis section, page 11, line 194-196 and results section, page 13, line 233-239. |
| 3. Are the conclusions supported by the data and do they address the research question presented in the Registered Report Protocol?   The manuscript must describe a technically sound piece of scientific research with data that supports the conclusions. The conclusions must be drawn appropriately based on the research question(s) outlined in the Registered Report Protocol and on the data presented.  Reviewer #1: No  Reviewer #2: Yes | Thank you for your answer. The conclusions of this manuscript respond to objectives of the original research protocol. These objectives were just adapted to make more meaning. The conclusions of this manuscript are based on the objectives of the manuscript. |
| 4. Have the authors made all data underlying the findings in their manuscript fully available?  The [PLOS Data policy](http://www.plosone.org/static/policies.action#sharing) requires authors to make all data underlying the findings described in their manuscript fully available without restriction, with rare exception (please refer to the Data Availability Statement in the manuscript PDF file). The data should be provided as part of the manuscript or its supporting information, or deposited to a public repository. For example, in addition to summary statistics, the data points behind means, medians and variance measures should be available. If there are restrictions on publicly sharing data—e.g. participant privacy or use of data from a third party—those must be specified.  Reviewer #1: No  Reviewer #2: Yes | Thanks for your answer. We made all data underlying the findings in this manuscript fully available by submitting the data base with the previous revision. |
| 5. Is the manuscript presented in an intelligible fashion and written in standard English?  *PLOS ONE* does not copyedit accepted manuscripts, so the language in submitted articles must be clear, correct, and unambiguous. Any typographical or grammatical errors should be corrected at revision, so please note any specific errors here.  Reviewer #1: No  Reviewer #2: Yes | Thanks for your answer. The manuscript has been read and edited by a native English speaker. |
| **Review comments to the authors** | |
| **Reviewer 1** | |
| An article seems more sophisticated and scientific.  However, its devised version does not change the results and their clinical meanings to improve outcomes on maternal and fetal health. | Thanks very much for your observation. We hope that our explanations help clarify some issues |
| The authors found that ionised Ca of the pregnant mothers seems to have impacts on fetal outcomes, including Apgar scores at one and five minutes after birth. However, the issues of neonates must be occurrence of events seen at longer observational periods of time, such as 7-day, 14-day, or 28-day mortality or IRDS. If neonates have lower Apgar score (AS) at birth, neonatologists could treat them properly and AS seems no effects on 7-, 14-,28-days mortality. | Thank you very much for your comment. Our manuscript presents results from a registered protocol. We perfectly agree that neonatal follow up periods extend to 28days, and our study did not look as far as that to see if other low calcium-related adverse outcomes could occur. So long term effects of low calcium were not our target (this has been included as a limit of this study). See page 25, line 421-424.  According our methods, the factors (were low calcium, low albumin and calcium supplementation in the mother), and our outcomes of interest were measured at term and birth. All selected foetal outcomes have been shown in literature to have potential negative effects on the health of the child in future. For example, low Apgar scores have been associated with poor neurological development and lower IQ (2,3), respiratory distress, hypothermia, feeding problems (4), higher neonatal mortality (5). Similar effects can be cited for low birth weight and others.  More interest is put on which maternal variables could be acted upon to reduce adverse maternal and foetal outcomes. We found out that the key determinant was the ionised calcaemic state, sometimes the calcium supplementation.  Children with poor Apgar scores could be resuscitated by neonatologist as stated by the reviewer and the course of evolution might not show any long term effects. But we feel that detecting a factor like the calcaemic state of the mother (and acting on it) could help reduce the number of children born with low Apgar score. The same goes for children with low birth weight (responsible for 60-80%) of neonatal deaths (6). Preventing the birth of babies with low Apgar scores and low birthweight is, therefore, better than having neonatologists ready to resuscitate these babies.  Moreover, our finding of the effect of ionised calcaemic state on hypertensive disease in pregnancy is a turning point. Acting only at this level prevents its occurrence and adverse maternal and foetal outcomes associated with hypertension in pregnancy (especially preeclampsia and eclampsia). |
| In conclusion, although the authors had tremendously a lot of time to study, the results seems no clinical impacts on neonates and this might be rejected. | Thanks for your comment. We hope that our explanations have convincing enough. Do maternal variables like calcaemic state, albuminaemia state and calcium supplementation affect maternal and foetal outcomes? Can these factors be acted upon to prevent the occurrence of adverse outcomes? We think the answer is yes. In this light, we believe our findings can contribute to improving maternal and feotal health. |
| **Reviewer 2** | |
| All comments raised by the first and second revision are addressed: Thanks for that and congratulation. | Thank you very much for your valuable contributions |

**References**

1.	Ajong AB, Kenfack B, Ali IM, Yakum MN, Aljerf L, Telefo PB. Hypocalcaemia and calcium intake in pregnancy: A research protocol for critical analysis of risk factors, maternofoetal outcomes and evaluation of diagnostic methods in a third-category health facility, Cameroon. PLoS One. 2020;15(11 November).

2.	Hassen TA, Chojenta C, Egan N, Loxton D. The association between the five-minute apgar score and neurodevelopmental outcomes among children aged 8−66 months in Australia. Int J Environ Res Public Health. 2021;18(12).

3.	Tweed EJ, Mackay DF, Nelson SM, Cooper SA, Pell JP. Five-minute Apgar score and educational outcomes: Retrospective cohort study of 751 369 children. Arch Dis Child Fetal Neonatal Ed. 2016;101(2):F121–6.

4.	Thavarajah H, Flatley C, Kumar S. The relationship between the five minute Apgar score, mode of birth and neonatal outcomes. J Matern Neonatal Med. 2018;31(10):1335–41.

5.	Cnattingius S, Norman M, Granath F, Petersson G, Stephansson O, Frisell T. Apgar Score Components at 5 Minutes: Risks and Prediction of Neonatal Mortality. Paediatr Perinat Epidemiol. 2017;31(4):328–37.

6.	Njim T, Atashili J, Mbu R, Choukem SP. Low birth weight in a sub-urban area of Cameroon: An analysis of the clinical cut-off, incidence, predictors and complications. BMC Pregnancy Childbirth. 2015;15(1).
