# Supplementary material for: Adverse maternofoetal outcomes associated with ionised calcaemia, total calcaemia, albuminaemia, and calcium supplementation in pregnancy: Analysis from a resource-limited setting
Source: PLoS One. 2022 Aug 1;17(8):e0271525. doi: 10.1371/journal.pone.0271525 (PMC9342720; doi:10.1371/journal.pone.0271525)
Supplement: Attachment — Submitted filename: Response to Reviewers R3.docx [file pone.0271525.s004.docx]

**Response to Reviewers**

| **Reviewer’s comments** | **Response** |
| --- | --- |
| This is revised article studying an association between adverse events of neonate and biochemical parameters of serum calcium and albumin concentration of pregnant mothers at 37 and over of pregnant period before delivering neonates. The authors found associations of maternal lower ionized calcium level <= 131 mmol/L and low birth weight, low birth length, low brachial circumference at birth, and low Apgar score at one and fifth minutes after birth. Moreover, they stated that calcium supplementation significantly improved the Apgar scores at the first and fifth minutes showing their results in Table 3. | Thank you for the review. Our article evaluates associations between hypocalcalcaemic states and maternofoetal outcomes. We have reviewed the title to fit the design of the study. In essence, our study evaluated associations which could be potential hypotheses for future longitudinal studies (for cause-effect relationships). This is recognized as a limit to our findings in the manuscript. See title and section on limits. |
| Form reading carefully their descriptions about evaluating maternal calcium supplementations during pregnant periods, it is unclear how much thy took calcium supplementation adding on dietary intake. The methodological problem that I am feeling is that daily total calcium intakes at late pregnant periods of meal and calcium supplementation must be calculated. Otherwise, it must be unclear which pregnant mother must be target to supplement calcium because she is deficient calcium intake. In addition, serum vitamin D concentrations and time durations of sunlight exposure also must be considered when which pregnant women is the target of calcium supplementations to prevent poor maternal or neonatal outcomes. Without disclosed above mentioned problems must be resolved, this article could not provide meaningful information to health care professionals. In conclusion, I would say that this revised article must not be accepted. | Thank you for your concern. Our objective in this manuscript was not to evaluate the calcium intake of the participants in the diet and supplements. As stated in response to reviewers 1, We have a manuscript evaluating calcium intake (in terms of calcium supplementation practices in pregnancy) and determines factors affecting this practice in pregnancy (given that it is recommended by the WHO to be systematic in LMIC for the prevention of hypertensive diseases in pregnancy). This was submitted on the 1st 0ct 2021 (PONE-D-21-31679).  Our key problem was to evaluate the relative strength of association that ionized and total calcaemic states could have on maternofoetal outcomes. In doing this we attached a simple question to see if there was an association between “haven taken calcium supplements in pregnancy” and maternofoetal outcomes. We were not looking for which women to supplement and which women not to supplement.  We understand that Vit D concentrations and exposure to sunlight contribute to providing active calcitriol for intestinal absorption of calcium. Vit D and sunlight exposure concur to increasing concentrations of calcitriol which increases intestinal calcium absorption. Therefore, we believe that to evaluate the association between calcaemic states and maternofoetal outcomes, there is no need to control for sunlight exposure and Vit D concentrations. This because the terminal point of Vit D concentrations and sunlight exposure is calcaemia. |
